# Which cognitive and linguistc factors influence phonological processing in adolescents?

**DOI:** 10.1590/2317-1782/20212020158

**Published:** 2021-10-18

**Authors:** Luciana Cássia de Jesus, Luciana Mendonça Alves, Vanessa de Oliveira Martins-Reis

**Affiliations:** 1 Programa de Pós-graduação em Ciências Fonoaudiológicas, Universidade Federal de Minas Gerais – UFMG - Belo Horizonte (MG), Brasil.; 2 Departamento de Fonoaudiologia, Universidade Federal de Minas Gerais – UFMG - Belo Horizonte (MG), Brasil.; 3 Departamento de Fonoaudiologia, Universidade de Brasília – UnB – Brasília (DF), Brasil.

**Keywords:** Language, Language Tests, Cognition, Mental Processes, Adolescents

## Abstract

**Purpose:**

To characterize phonological processing of adolescents and to identify language skills and cognitive functions that influence their age group.

**Methods:**

83 typical adolescents aged from 11 to 16 years of age participated in the research. Phonological awareness tests, rapid automatic naming, neuropsychological assessment and reading were used. Descriptive analysis and linear regression were carried out with a 5% significance level.

**Results:**

Regarding phonological processing, a lower performance was found in the phonemic segmentation task, longer times for object naming and performance as were expected for working memory of the age range. There was a reciprocal association between rapid naming of objects, letters and working memory, between rapid naming of letters and phonological awareness. Executive functions and attention influence working memory and phonological awareness. Semantic episodic verbal memory influenced working memory and reading, phonological awareness.

**Conclusion:**

The performance in phonological processing was influenced by linguistic and cognitive skills which suggests they are still improving in adolescents.

## INTRODUCTION

The development of written language is influenced by among other factors the ability to process language automatically, from its sound information, called "phonological processing"^(1)^.

Several studies have shown that phonological processing skills (phonological awareness, working memory and rapid automatic naming) are predictors of written language development, due to their relevance in the ability to process, retain and retrieve information^(2,3,4)^ for writing and reading success, both in terms of decoding^(5)^ and comprehension^(6)^.

It is expected that performance in phonological processing will increase through adolescence^(7)^ due to an increase in age, vocabulary and cognitive abilities, with information being accessed with greater speed and accuracy and with less data loss, ^(8,9)^, consequently more cognitive and attentional resources will be available for reading which is an important aspect of reading comprehension^(10,11)^.

According to studies phonological awareness allows a person to consciously perceive and manipulate the sound information of words at various levels: intrasyllabic, syllabic and phonemic^(4)^. It is important in the initial acquisition of reading and in the decoding of unknown regular words, as it allows for establishing a relationship between phoneme and grapheme^(5)^.

Working memory on the other hand is responsible for retrieving orthographic and phonological data in the mental lexicon, while graphophonemic associations are performed. It participates in the learning of new words, syntactic analysis and reading and language comprehension^(6)^.

Rapid naming capability allows for quick access to information which is an important aspect for reading fluency, through the involvement of various cognitive resources^(10)^. When information is accessed quickly and accurately in the mental lexicon, more cognitive and attentional resources will be available for reading^(11)^.

Studies that have already investigated the interaction of linguistic and cognitive skills with phonological processing show a reciprocity relationship^(2,12)^. The appropriation and increase in reading and writing proficiency also drive and can explain the development of higher levels of phonological processing^(2,12)^; however studies investigating the performance of phonological processing of adolescents as a response variable and explained by linguistic-cognitive skills are still scarce.

This study aims to characterize phonological processing of adolescents and identify language skills and cognitive functions that influence this age group.

## METHOD

This study has an analytical observational cross-sectional design and was approved by the Research Ethics Committee of the Federal University of Minas Gerais 1,722.230. Adolescents and their guardians signed a consent form to participate in the study.

### Participants

The sample was non-probabilistic and based on convenience. Adolescents from two public secondary elementary schools in Belo Horizonte were invited to participate in the study and 110 adolescents showed interested in being part of the study sample. Those whose parents reported typical development in the anamnesis questionnaire were selected. The exclusion criterion adopted were any presence or evidence of neurological, psychiatric, cognitive and learning alterations, when reported during the filling out of the anamnesis questionnaire, incorrect hearing and visual alterations and the non-completion of the test. After analyzing the anamnesis questionnaires, four adolescents were excluded because of diagnoses of developmental disorders as reported by their parents. The initial sample consisted of 106 adolescents with typical development. During the research, 23 adolescents dropped out of the study and did not complete the assessments, therefore the final sample consisted of 83 adolescents of both sexes enrolled in schools normally from sixth to ninth grades. Normal students in elementary schools were selected and the sample age ranged from 11 to 16 years of age.

### Instruments

- Anamnesis questionnaire - prepared by the researchers with questions related to the adolescent's health history and development.
- Phonological awareness test of the Battery for the Assessment of Written Language and its Disorders – BALESC^(13,14)^: The test consists of syllabic and phonemic manipulation tasks. Studies show that linguistic and academic experiences present when entering school favor acquisitions at the syllabic level of phonological awareness, enabling good performance at this level in early school years, making it internalized in elementary school. Furthermore as phonemic awareness is more closely related to reading^(5)^, it was decided to use only phonemic manipulation tasks, namely: segmentation, subtraction and inversion. Test stimuli were recorded to avoid interference.
- RAN automatic naming speed test – rapid automatized naming.^(15)^: boards with visual stimuli letters and objects randomly arranged in ten columns and five lines were used.
- Brief Neuropsychological Assessment Instrument – NEUPSILIN^(16)^: to assess attention, inverse counting and digit repetition tests were carried out. Memory was assessed using digit and word span tests, recognition, immediate and delayed recall, long-term semantic memory, short-term visual memory, and prospective memory. To assess oral language, naming, repetition, comprehension, inference processing and automatic language tasks were performed. Executive functions were assessed by solving oral problems and verbal fluency. According to the correction proposed by the authors of the instrument, the results of working memory, episodic-semantic verbal memory, attention and oral language were also presented in a qualitative way, classified as adequate or inadequate according to the standards referred to by the test.
- Textual reading fluency and comprehension test: The instrument proposed by Gentilini et al.^(17)^ was used, consisting of a text and ten literal and inferential questions. The instrument was designed to assess adolescents in secondary elementary school. The construction of the instrument involved several steps, including selection and analysis by expert judges and computational analysis, so that it met the criteria for linguistic assessment of adolescents.

### Procedures

Participants were evaluated in a quiet room at the school, in 30 minute sessions a day, with an average total of two hours. Firstly, the anamnesis questionnaire was sent to the parents, together with the terms of authorization to be able to participate in the study, which was later collected to assist in the selection of adolescents according to the inclusion and exclusion criteria.

Afterwards the selected adolescents were submitted for testing as described above, following the rules as defined by previous research and published manuals. Only the reading assessment was applied collectively.

### Data analysis

The data was entered into an Excel® spreadsheet. In the descriptive analysis of qualitative variables, absolute and relative frequencies were used, while in the description of quantitative variables measures of position, central tendency and dispersion were used.

To verify the effect of cognitive and linguistic variables on phonological processing, linear regression with robust standard errors was used for the covariance matrix of the estimated coefficients and the robust estimator HC (heteroskedasticity and consistent autocorrelation) was used to estimate the covariance matrix.

For selection of variables, the Stepwise method (Forward and Backward) was used. Thus, using the Forward method, a univariate analysis was performed, which consisted of adjusting a linear regression with robust standard errors, for each of the variables separately. Variables with p-value less than 0.25 were selected for multivariate analysis. In the multivariate analysis, a model with all selected variables was adjusted, then the presence of multicollinearity between them was verified. For this purpose, the VIF (Variance Inflation Factor) statistic was used, and the variables with a VIF value greater than five were removed from the model. Then, the Backward method was carried out, a procedure used to remove in turn, the variable with the highest p-value which was repeated until only the significant variables remained in the model. For the Backward method, a significance level of 5% was adopted. Version 3.4.3 of the R software was used in the analyses.

The variables reading fluency, text comprehension, episodic-semantic verbal memory attention, executive functions and oral language tasks were considered explanatory for the response variables: naming time in objects and letters, working memory and phonological awareness, which were also explanatory of the others in the regression analysis models.

## RESULTS

**Figure 1:**
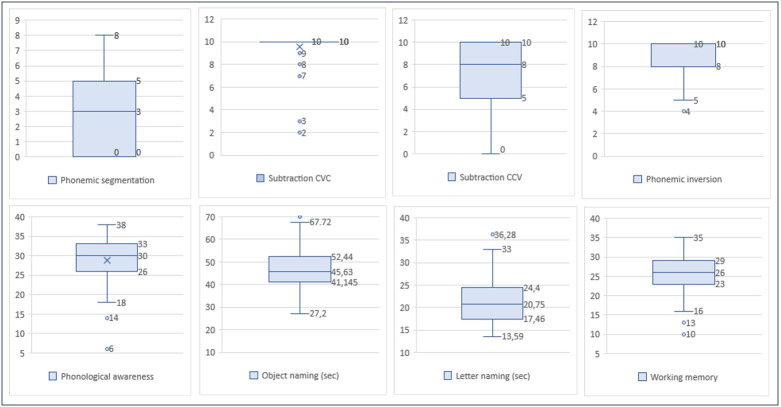
Measures of central tendency and dispersion of Phonological Processing tasks

**Figure 2:**
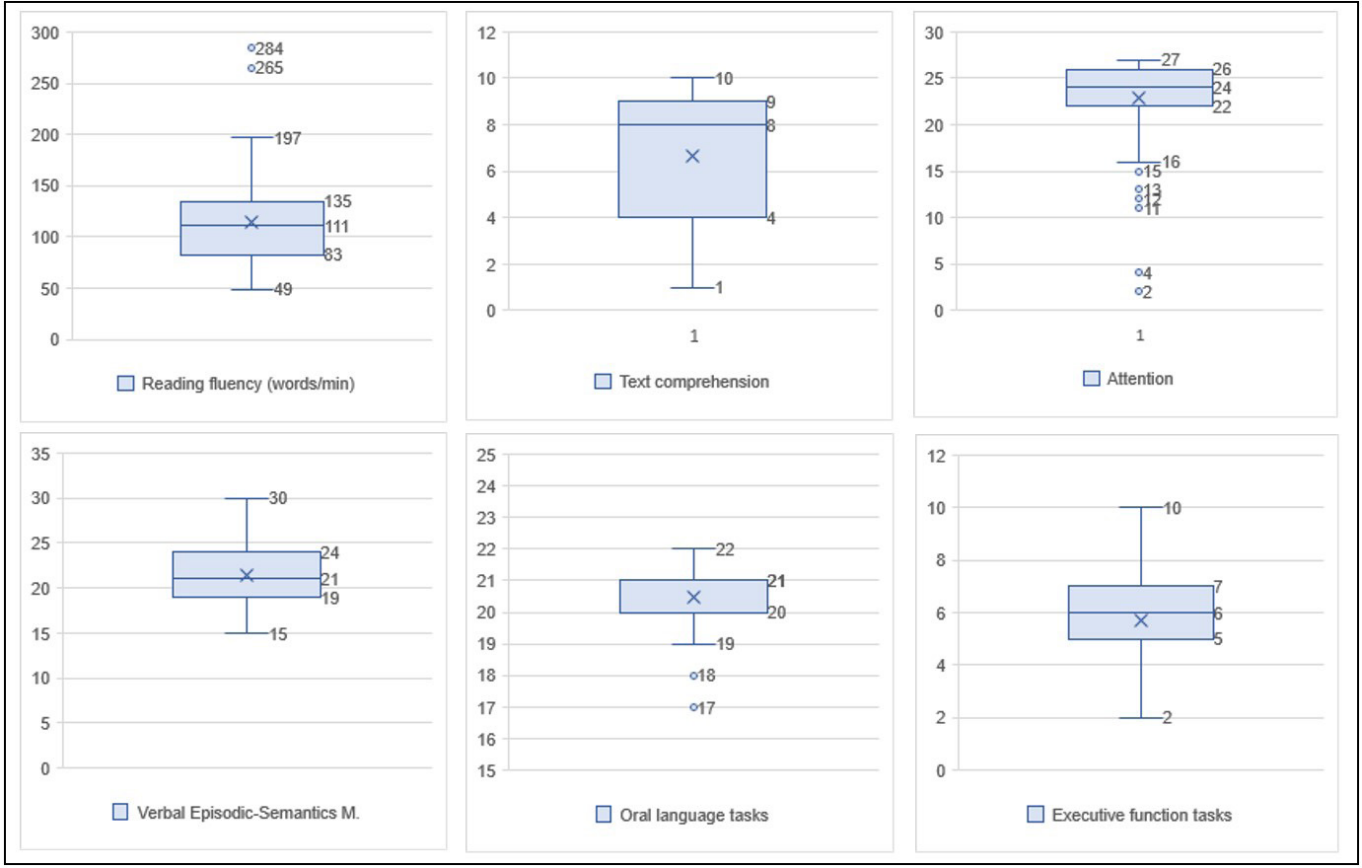
Measures of central tendency and dispersion of cognitive and linguistic variables

The measures of central tendency and dispersion of clinical variables were presented in Figures 1 and 2.

**Caption**: cvc = consoant-vowel-consoant; ccv = consoant-consoant-vowel; seg = seconds

**Caption**: min = minute; M. = memory

Regarding the classification of the participants' performance in neuropsychological assessment tasks, it was found that 82.7% (n = 62) had adequate performance in working memory, 87.8% (n = 65) in attention, 65, 3% (n = 49) in episodic-semantic verbal memory and 85.3% (n = 64) in oral language tasks. Oral language was assessed through the tasks of naming, repetition, comprehension and processing of inferences and automatic language. The term “appropriate oral language” was used when the overall score was within the normal range determined by the test.

### Factors associated with naming time for objects and letters

Table 1 shows the results of the univariate analysis between the cognitive and linguistic factors studied and the rapid naming of objects and letters. In general, quick naming showed a statistically significant association with almost all analyzed variables.

**Table 1:** Factors associated with fast naming of objects and letters - univariate analysis

	**Object naming**				**Letter naming**			
**Variable**	***β***	**S.E.** 1	**C.I - 95%^1^**	***p* ^1^**	***β***	**S.E.^1^**	**C.I - 95%^1^**	***p* ^1^**
Working memory	-0.97	0.34	[-1.64; -0.30]	**0.005**	-0.11	0.10	[-0.30; 0.09]	0.275
Operating memory (adequate)	-	-	-	-	-	-	-	-
Working memory (inappropriate)	12.37	4.93	[2.71; 22.04]	**0.012**	2.41	1.22	[0.02; 4.80]	**0.048**
Phonological awareness	-0.62	0.16	[-0.93; -0.31]	**< 0.001**	-0.28	0.06	[-0.39; -0.16]	**< 0.001**
Letter naming	1.45	0.25	[0.97; 1.93]	**< 0.001**	**-**	**-**	**-**	**-**
Object naming	-	-	-	**-**	0.24	0.04	[0.16; 0.32]	**< 0.001**
Reading fluency	-0.12	0.03	[-0.18; -0.06]	**< 0.001**	-0.05	0.01	[-0.07; -0.03]	**< 0.001**
Text comprehension	-2.01	0.52	[-3.03; -0.98]	**< 0.001**	-0.73	0.18	[-1.09; -0.37]	**< 0.001**
Episodic-semantic verbal memory	-0.33	0.42	[-1.14; 0.49]	0.430	-0.24	0.15	[-0.54; 0.06]	0.124
Verbal episodic-semantic M.(adequate)	-	-	-	-	-	-	-	-
Episodic-semantic verbal M. (inappropriate)	2.80	2.96	[-3.00; 8.61]	0.343	2.55	1.25	[0.10; 5.00]	**0.042**
Oral language tasks	-4.49	1.73	[-7.89; -1.09]	**0.010**	-1.27	0.57	[-2.38; -0.16]	**0.025**
Oral language tasks (appropriate)	-	-	-	-	-	-	-	-
Oral language tasks (inadequate)	8.59	5.38	[-1.96; 19.13]	0.111	2.43	1.48	[-0.48; 5.34]	0.101
Executive functions tasks	-2.44	0.61	[-3.64; -1.24]	**< 0.001**	-0.72	0.26	[-1.23; -0.21]	**0.006**
Attention	-0.62	0.33	[-1.28; 0.03]	**0.063**	-0.16	0.08	[-0.31; -0.01]	**0.037**
Attention (appropriate)	-	-	-	-	-	-	-	-
Attention (inappropriate)	9.62	5.77	[-1.68; 20.92]	0.095	1.82	1.22	[-0.58; 4.21]	0.137

1 Calculated using the HC estimator;

**Caption**: *β* = regression coefficient; S.E. = standard error; C.I = confidence interval; M. = memory; p<0.05

The final multivariate regression model (Table 2) showed that each additional point in the working memory score leads to an average reduction of -0.72 seconds in object naming time and that an increase of 1 second in the naming of objects. letters lead to an average increase of 1.05 seconds in object naming time. Regarding the final model of the multivariate analysis for letter naming (Table 2), it can be found that each point that increases the performance in phonological awareness and each increase of one word in the total number of words read per minute leads to a reduction of - 0.11 and 0.02 seconds respectively in letter naming time. Furthermore for each 1s increase in object naming time, an average increase of 0.18s in letter naming time occurs.

**Table 2:** Factors associated with rapid naming of objects and letters - multivariate analysis

**Variable**	**Object naming**								**Letter naming**			
	**Initial model**				**Final model**				**Initial model**			
	***β***	**S.E.** 1	**C.I - 95%^1^**	***p* ^1^**	***β***	**S.E.^1^**	**C.I - 95%^1^**	***p* ^1^**	***β***	**S.E.^1^**	**C.I - 95%^1^**	***p* ^1^**
Working memory	-0.80	0.33	[-1.44; -0.15]	**0.015**	-0.72	0.26	[-1.24; -0.21]	**0.006**				
Phonological awareness	0.12	0.17	[-0.21; 0.44]	0.489	-	-	-	-	-0.12	0.08	[-0.27; 0.03]	0.105
Operating memory (adequate)									-	-	-	-
Working memory (inappropriate)									-0.47	1.21	[-2.85; 1.91]	0.698
Episodic-semantic verbal memory									-0.19	0.15	[-0.48; 0.09]	0.182
Episodic-semantic verbal memory (adequate)	-	-	-	-	-	-	-	-				
Episodic-semantic verbal memory (inappropriate)	-4.30	2.53	[-9.25; 0.66]	0.089	-	-	-	-				
Letter naming	1.11	0.23	[0.65; 1.56]	**<0.001**	1.05	0.20	[0.65; 1.44]	**<0.001**				
Object naming									0.18	0.05	[0.08; 0.28]	**<0.001**
Reading fluency	-0.04	0.02	[-0.08; 0.00]	**0.032**	-0.04	0.02	[-0.07; 0.00]	**0.051**	-0.02	0.01	[-0.04; 0.00]	**0.016**
Text comprehension	-0.77	0.44	[-1.62; 0.09]	0.078	-0.73	0.38	[-1.48; 0.03]	**0.058**	-0.10	0.22	[-0.53; 0.34]	0.667
Oral language tasks	-1.51	0.99	[-3.46; 0.43]	0.127	-	-	-	-	-0.06	0.46	[-0.96; 0.84]	0.897
Executive functions tasks	0.04	0.62	[-1.18; 1.25]	0.951	-	-	-	-	-0.01	0.26	[-0.53; 0.50]	0.958
Attention	0.10	0.22	[-0.34; 0.53]	0.668	-	-	-	-	0.11	0.10	[-0.08; 0.31]	0.244
R^2^	46.21%				48.16%				37.21%			
VIF Maximum	1.79				1.36				1.67			

1 HC estimator;

**Caption**: β = regression coefficient; S.E. = standard error; C.I = confidence interval; p<0.05

### Factors associated with working memory

Table 3 shows the results of the univariate analysis of the cognitive and linguistic factors studied and working memory (continuous and categorical). There was a statistically significant association between working memory and the following variables: phonological awareness (continuous), object naming (continuous and categorical), textual comprehension (categorical), episodic-semantic verbal memory (continuous), oral language (continuous), executive functions (continuous and categorical) and attention (continuous and categorical).

**Table 3:** Factors associated with working memory scores and performance rating - univariate analysis

**Variable**	**Score**				**Performance rating**				
	***β***	**S.E.** 1	**C.I - 95%^1^**	***p* ^1^**	***β***	**S.E.^1^**	**C.I - 95%^1^**	***p* ^1^**	**O.R.**
Letter naming	-0.12	0.11	[-0.34; 0.1]	0.297	0.10	0.06	[-0.01; 0.21]	0.074	1.11
Phonological awareness	0.17	0.08	[0.01; 0.33]	**0.041**	-0.06	0.04	[-0.14; 0.01]	0.113	0.94
Object naming	-0.17	0.05	[-0.27; -0.07]	**0.001**	0.08	0.02	[0.03; 0.12]	**0.001**	1.08
Reading fluency	0.02	0.01	[-0.01; 0.04]	0.190	-0.01	0.01	[-0.03; 0.01]	0.274	0.99
Text comprehension	0.41	0.22	[-0.02; 0.83]	0.060	-0.26	0.13	[-0.51; -0.01]	**0.043**	0.77
Episodic-semantic verbal memory	0.50	0.13	[0.25; 0.74]	**<0.001**	-0.17	0.10	[-0.37; 0.04]	0.107	0.85
Episodic-semantic verbal memory (adequate)	-	-	-	-	-	-	-	-	-
Episodic-semantic verbal memory (inappropriate)	-3.28	1.15	[-5.54; -1.02]	**0.004**	0.97	0.62	[-0.25; 2.19]	0.118	2.64
Oral language tasks	1.70	0.82	[0.09; 3.3]	**0.039**	-0.69	0.38	[-1.44; 0.06]	0.072	0.50
Oral language tasks (adequate)	-	-	-	-	-	-	-	-	-
Oral language tasks (inappropriate)	-3.14	2.36	[-7.76; 1.48]	0.183	2.28	0.73	[0.86; 3.7]	**0.002**	9.77
Executive functions tasks	1.35	0.25	[0.87; 1.83]	**<0.001**	-0.49	0.18	[-0.84; -0.14]	**0.006**	0.61
Attention	0.52	0.10	[0.33; 0.72]	**<0.001**	-0.19	0.05	[-0.28; -0.1]	**<0.001**	0.83
Attention (adequate)	-	-	-	-	-	-	-	-	-
Attention (inappropriate)	-6.43	1.98	[-10.31; -2.55]	**0.001**	1.60	0.76	[0.11; 3.1]	**0.035**	4.98

1 Calculated using the HC estimator;

**Caption**: *β* = regression coefficient; S.E. = standard error; C.I = confidence interval; O.R. = *odds ratio*, *p*<0,05

The final multivariate regression model (Table 4) showed that for each 1-second increase in the time for naming objects there is an average reduction of 0.09 in the working memory score. The addition of one unit in the episodic-semantic verbal memory, executive functions and attention score leads to a respective increase of 0.33, 0.83 and 0.34 points in the working memory score. Although in the final model the VIF was low, the textual comprehension variable was correlated with almost all the explanatory variables in the final model, such as attention, executive functions, episodic-semantic verbal memory and time for naming objects. A model with correlated explanatory variables leads to the occurrence of multicollinearity, which causes the inversion of the estimated coefficient sign (β), consequently it was necessary to remove the textual understanding variable from the final model.

**Table 4:** Factors associated with working memory scores and performance rating - multivariate analysis

**Variable**	**Score**								**Performance rating**	
	**Initial model**				**Final model**				**Initial model**	
	***β***	**S.E.** 1	**C.I - 95%^1^**	***p* ^1^**	***β***	**S.E.^1^**	**C.I - 95%^1^**	***p* ^1^**	***β***	**S.E.^1^**
Phonological awareness	-0.04	0.07	[-0.18; 0.09]	0.397	-	-	-	-	0.05	0.05
Letter naming									-0.06	0.11
Object naming	-0.12	0.04	[-0.19; -0.05]	**0.001**	-0.09	0.04	[-0.17; -0.01]	**0.034**	0.08	0.04
Episodic-semantic verbal M.	0.32	0.09	[0.14; 0.51]	**<0.001**	0.33	0.09	[0.14; 0.51]	**<0.001**		
Reading fluency	-0.01	0.01	[-0.03; 0.01]	0.369	-	-	-	-	0.00	0.01
Text comprehension	-0.31	0.17	[-0.64; 0.03]	**0.060**	-	-	-	-	0.03	0.19
Oral language tasks	0.50	0.54	[-0.57; 1.56]	0.344	-	-	-	-	-0.39	0.48
Executive functions tasks	1.00	0.26	[0.48; 1.51]	**<0.001**	0.83	0.25	[0.34; 1.32]	**0.001**	-0.53	0.35
Attention	0.37	0.08	[0.21; 0.54]	**<0.001**	0.34	0.08	[0.2; 0.49]	**<0.001**	-0.17	0.06
R^2^	45,39%				43,47%					42,32%
VIF Maximum	1.59				1.17					2.06

1 Calculated using the HC estimator;

**Caption**: *β* = regression coefficient; S.E. = standard error; C.I = confidence interval; O.R. = *odds ratio*; M. = memory; *p*<0,05

Regarding the final multivariate regression model for the classification of working memory performance (Table 4), it was found that an increase in one second in object naming time increases by 1.07 times the chances of working memory being classified as inadequate. On the other hand, each point that increases in the attention score decreases the chance of working memory being classified as inadequate by 15%.

### Factors associated with phonological awareness

The results of the univariate and multivariate regression analysis for phonological awareness are shown in Table 5. There is a statistically significant association between phonological awareness and the time of naming letters and objects, working memory, oral language, reading fluency, comprehension of reading, executive functions and attention.

**Table 5:** Factors associated with phonological awareness - uni and multivariate analysis

**Variable**	**Univariate analysis**				**Multivariate analysis**								
	***β***	**S.E.** 1	**C.I - 95%^1^**	***p* ^1^**	**Initial model**				**Final model**			
					***β***	**S.E.^1^**	**C.I - 95%^1^**	***p* ^1^**	***β***	**S.E.^1^**	**C.I - 95%^1^**	***p* ^1^**	
letter naming	-0.50	0.12	[-0.74; -0.27]	**< 0.001**	-0.21	0.13	[-0.47; 0.06]	0.125	-0.37	0.10	[-0.57; -0.17]	**< 0.001**	
Operating memory (adequate)	-	-	-	-									
Working memory (inappropriate)	-2.82	1.65	[-6.06; 0.42]	0.088									
working memory	0.28	0.11	[0.07; 0.50]	**0.010**	-0.12	0.16	[-0.44; 0.19]	0.444	-	-	-	-	
object naming	-0.18	0.05	[-0.28; -0.09]	**< 0.001**	0.00	0.06	[-0.11; 0.12]	0.947	-	-	-	-	
reading fluency	0.05	0.01	[0.03; 0.07]	**< 0.001**	0.02	0.01	[-0.01; 0.04]	0.198	-	-	-	-	
text comprehension	1.06	0.23	[0.62; 1.51]	**< 0.001**	0.43	0.24	[-0.05; 0.91]	0.077	-	-	-	-	
episodic-semantic verbal memory	0.09	0.17	[-0.25; 0.43]	0.592									
Episodic-semantic verbal memory (adequate)	-	-	-	-									
Episodic-semantic verbal memory (inappropriate)	-0.03	1.52	[-3.02; 2.95]	0.983									
oral language tasks	1.89	0.64	[0.64; 3.15]	**0.003**	0.65	0.64	[-0.60; 1.90]	0.310	-	-	-	-	
Oral language tasks (adequate)	-	-	-	-									
Oral language tasks (inappropriate)	-4.28	2.40	[-8.97; 0.42]	0.074									
Executive functions tasks	1.40	0.40	[0.62; 2.19]	**< 0.001**	0.71	0.43	[-0.13; 1.55]	0.097	0.87	0.42	[0.04; 1.70]	**0.039**	
Heads up	0.50	0.09	[0.32; 0.69]	**< 0.001**	0.34	0.12	[0.11; 0.57]	**0.004**	0.37	0.10	[0.17; 0.57]	**< 0.001**	
Attention (adequate)	-	-	-	-									
Attention (inappropriate)	-6.04	1.85	[-9.67; -2.42]	**0.001**									
R^2^					27.38%				26.36%			
VIF Maximum					2.09				1.11			

1 HC estimator;

**Caption**: β = regression coefficient; S.E. = standard error; C.I = confidence interval; p<0.05

The final multivariate regression model showed that a one-second increase in time in letter naming reduces -0.37 points in the phonological awareness score; however for each unit that increases in the score of executive functions and attention, an increase in phonological awareness performance on average occurs of 0.87 and 0.37 respectively.

## DISCUSSION

Studies^(3)^ show the importance of phonological processing in the initial learning of reading and writing in children. However, there are still gaps in knowledge about the performance of older people (who have already reached a certain level of cognitive development and learning) in phonological processing and the factors that interfere with such performance. Thus, this study sought to characterize the phonological processing skills in adolescents and point out which cognitive and linguistic factors influence the development of these skills.

Regarding cognitive performance, the adolescents selected did not present linguistic or cognitive impairment, therefore they were considered typically developed adolescents. Their expected performance was adequate, as observed in the majority of the samples. In this study, adolescents showed a performance similar to that of young adults with a higher level of education in a previous survey^(18)^. No ceiling effect was found, as occurred in the present study, which suggests that the cognitive performance of adolescents is on its way to the peak of it's development.

Descriptive data for rapid naming showed that the shortest naming time was in letters and the longest naming time in objects, corroborating the data found in previous studies^(12)^. According to other studies, knowledge of letters and digits is quickly automated, which makes naming agile and reduces the time^(6)^. As for the naming of colors and objects, studies show that concepts and semantic information are accessed prior to naming, in addition to stimuli having greater articulation extension and complexity, which increases the time taken to complete the test^(12)^.

Regarding the performance of working memory, most participants, revealed typical development and being in the age group in which the expansion of working memory is found, presented adequate performance, according to the scores proposed in the NEUPSILIN test^(16)^. It is known that an increase in age and school year expands working memory capacities, enabling the handling of a greater amount of information simultaneously^(8,9)^.

The adolescents' performance in phonological awareness was better in the consonant-vowel-consonant (CVC) subtraction task, followed by the consonant-consonant-vowel (CCV) inversion and subtraction tasks. In the phonemic segmentation task, they had the lowest average of correct answers, showing that this was the one with the highest degree of difficulty for the evaluated sample. Another study which evaluated elementary school students from 1st to 5th grades with phonemic segmentation and subtraction tasks, found better performance in the CVC and CCV subtraction tests^(19)^. In the phonemic segmentation test and a lower average of correct answers was found. The study did not carry out the phonemic inversion task.

In another study^(20)^, in which three studies with adolescents and adults were analyzed, there was a low number of correct answers in the segmentation task, even for people proficient in reading. According to the study in question, after reading development, there may be less need to analyze a word from its phonological structure, so there would be a decline in such ability due to disuse. The second hypothesis presented suggested that when acquiring reading proficiency people acquire other strategies, such as word analysis at the orthographic level. Finally, the study indicated that explicit training is necessary for the development of phonemic awareness. Naturally a person's attention is directed to larger sound structures, such as rhyme and alliteration, the perception of phonemes is developed with training. Therefore, individuals with little stimulation would have a lower level of phonemic awareness and this would not be a condition that would prevent the achievement of high levels of reading ability.

It is also important to emphasize that phonemes are abstract units devoid of meaning. During phonemic segmentation an adolescent manipulates phonemes without the support of the acoustic information of the word and directs attention to its structural form, not its meaning. This increases the degree of difficulty of the task, justifying the low performance of adolescents^(19)^. There was no ceiling effect among adolescents, which suggests that this audience is in a phase of phonemic awareness improvement and that the difficulty in handling isolated phonemes persisted in the studied sample.

The results of the linear regression analysis showed that phonological processing was influenced by linguistic skills and cognitive functions, in addition to mutual interference between phonological processing skills. It was found that working memory, time for naming letters and objects, phonological awareness and executive functions were more significant in explaining the adolescents' performance in phonological processing.

Rapid object naming was influenced more by working memory and letter naming. The time for naming letters was influenced more by performance in phonological awareness and time for naming objects. Adolescents' performance in working memory and phonological awareness was better explained by executive functions.

The rapid naming of visual stimuli demands the involvement of various attentional memory processes, perceptual, conceptual, lexical and articulatory processes^(21)^. All these processes work in harmony to provide agility and accuracy in all naming tasks, which suggests the influence of the performance of one naming task over another. In the sample the association between the times of naming objects and letters was found. People who require more time in naming non-alphanumeric stimuli tend to spend more time in naming alphanumeric stimuli, and the inverse relationship is also present.

Object naming time was also influenced by working memory. So as the working memory score increases, the object naming time decreases. Due to the characteristics of the stimuli present in the RAN test, it was found that object naming has a semantic basis and greater phonological extension and complexity than letter naming, which has a phonological basis and less articulatory extension and complexity^(22)^. In addition to these facts, despite the fact that both activities are automatic, there is also the fact that the characteristic of naming letters is an automated process first in relation to naming objects^(22)^. The naming of objects required more of the working memory resources so that the stimuli were retrieved and kept in an accessible form in memory while they were named, which was not significant in the naming of letters, which required less effort and performance of the mnemonic process^(6,23)^.

Conversely as the time taken to perform the naming task increases there is a tendency to reduce the working memory score, increasing the chances of performance being classified as inadequate for some adolescents. As the naming of objects requires working memory resources^(21)^, the increase in the execution time of the naming test may reflect damage to mnemonic resources. There was a mutual influence between fast object naming and working memory.

Another relevant process for rapid naming was phonological awareness. It was found that the better the performance of adolescents in phonological awareness the better their agility in naming letters. In such a task, phonological manipulation is required throughout the test, so the handling of phonological representations with ease proved to be important to aid greater naming speed^(24)^.

According to the results of the study, adolescents who performed the naming tasks in a shorter time showed better performance in other tasks. It is worth noting that their agility in performing tasks is a relevant factor for their cognitive and linguistic performance.

The executive functions, responsible for regulating the information and behavior process^(25)^, were also significant in explaining the adolescents' performance in phonological awareness and working memory, contributing to elevated scores in such skills.

Due to control of their actions^(25)^, executive functions are relevant during phonemic manipulation tasks. The great importance of this ability is even more evident in studies of individuals with ADHD. According to the researchers, such individuals present alterations in language as a result of deficits in executive functions, leading to impairment in phonological processing^(6)^. The authors saw that students with ADHD performed poorly in phonological awareness and working memory when compared to normal students. Therefore, the data from this study corroborates existing studies by demonstrating that executive functions are relevant for the execution of the phonological awareness task.

Concerning the association of executive functions with working memory, this study used verbal fluency and problem solving tasks to assess executive functions. These tasks require the invocation and manipulation of information to be completed, and when relating words quickly or solving problems easily, there is less overload on the mnemonic system^(25)^. Good performance of executive functions is correlated positively with working memory. The results showed that among adolescents with good performance in executive function, there is a tendency to reduce the chances of being classified as having inadequate performance in working memory.

Another cognitive function that influenced the performance of phonological processing was attention, which is necessary to focus on the relevant stimulus and inhibit distractors. Thus, the storage capacity depends on maintaining the focus on the necessary stimulus, which suggests a relationship between attention and memory^(9)^.

In agreement with this hypothesis, in another study researchers found that in the presence of altered attention, memory is also compromised^(6)^. Among the adolescents evaluated in this study, attention was revealed as one of the determining factors for the performance of working memory.

Several studies have investigated phonological processing in children; however there is a need to expand this research with adolescents in order to deepen our knowledge about these skills in this target audience and the variables that influence them, in order to collaborate with clinical practice and the selection and development of assessment instruments suitable for adolescents.

The way in which this research sample was selected constituted a limitation for the generalization of the data. Although the sample is small, the data from this study may be reproduced in a larger sample, due to the low values of β and standard error obtained in the analyses. It is suggested to continue studies with adolescents in order to further investigate those already carried out.

This study showed that the clinicians should be aware of linguistic skills and cognitive functions that influence phonological processing, as they can impact the therapeutic processes.

## CONCLUSION

We concluded that for adolescents evaluated the automatic naming of letters was performed in a shorter time compared to the automatic naming of objects, and that there was a lower average of correct answers in the phonemic segmentation task. The phonological processing skills were related to each other. Executive functions and attention were more significant in explaining information sound processing skills, compared to reading and episodic-semantic verbal memory, which were less relevant.
