# Supplementary material for: Which cognitive and linguistc factors influence phonological processing in adolescents?
Source: Codas. 2021 Oct 18;34(1):e20200158. doi: 10.1590/2317-1782/20212020158 (PMC9886099; doi:10.1590/2317-1782/20212020158)
Supplement: Translation

# Translation: Quais fatores cognitivos e linguísticos influenciam o processamento fonológico em adolescentes?

**Authors:** Luciana Cássia de Jesus, Luciana Mendonça Alves, Vanessa de Oliveira Martins-Reis

**Affiliations:** 1 Programa de Pós-graduação em Ciências Fonoaudiológicas, Universidade Federal de Minas Gerais – UFMG - Belo Horizonte (MG), Brasil.; 2 Departamento de Fonoaudiologia, Universidade Federal de Minas Gerais – UFMG - Belo Horizonte (MG), Brasil.; 3 Departamento de Fonoaudiologia, Universidade de Brasília – UnB – Brasília (DF), Brasil.

**Keywords:** Linguagem, Testes de Linguagem, Cognição, Processos Mentais, Adolescentes

## Abstract

**Objetivo:**

Caracterizar o processamento fonológico em adolescentes e identificar habilidades linguísticas e funções cognitivas que o influenciam nessa faixa etária.

**Método:**

Participaram 83 adolescentes típicos de 11 a 16 anos. Utilizaram-se os testes de consciência fonológica, nomeação automática rápida, avaliação neuropsicológica e leitura. Foi realizada a análise descritiva e a regressão linear com nível de significância de 5%.

**Resultados:**

Em relação ao processamento fonológico, observou-se menor desempenho na tarefa de segmentação fonêmica, maior tempo de nomeação de objetos e desempenho dentro do esperado para a idade em memória operacional. Verificou-se associação recíproca entre nomeação rápida de objetos, de letras e memória operacional, entre nomeação rápida de letras e consciência fonológica. As funções executivas e a atenção influenciaram a memória operacional e a consciência fonológica. A memória verbal episódica semântica influenciou a memória operacional e, a leitura, a consciência fonológica.

**Conclusão:**

O desempenho no processamento fonológico foi influenciado por habilidades linguísticas e cognitivas e sugere ainda estar em aprimoramento na adolescência.

## INTRODUÇÃO

O desenvolvimento da linguagem escrita é influenciado, dentre outros fatores, pela capacidade de processar a língua de forma automática, a partir de suas informações sonoras, denominada “processamento fonológico” ^(1)^.

Diversos estudos apontam que as habilidades do processamento fonológico (consciência fonológica, memória operacional e nomeação automática rápida) são preditoras do desenvolvimento da linguagem escrita, devido à relevância para a capacidade de processar, reter e resgatar a informação^(2,3,4)^ para o sucesso da escrita e da leitura, tanto no nível da decodificação^(5)^ quanto da compreensão^(6)^.

Espera-se que o desempenho no processamento fonológico se amplie na adolescência^(7)^, em razão do aumento da idade, do vocabulário e das capacidades cognitivas, pois as informações são acessadas com maior velocidade e precisão e menor perda de dados ^(8,9)^, consequentemente mais recursos cognitivos e atencionais estarão disponíveis para a leitura, aspecto importante para a compreensão de leitura^(10,11)^.

De acordo com a literatura, a consciência fonológica possibilita que o indivíduo perceba e manipule, de forma consciente, as informações sonoras das palavras em vários níveis: intrassilábico, silábico e fonêmico^(4)^. É importante na aquisição inicial da leitura e na decodificação de palavras regulares desconhecidas, na medida em que permite estabelecer a relação entre fonema e grafema^(5)^.

Já a memória operacional é responsável por recuperar os dados ortográficos e fonológicos no léxico mental, enquanto é realizada a associação grafofonêmica. Participa do aprendizado de palavras novas, da análise sintática e da compreensão leitora e de linguagem^(6)^.

A capacidade de nomeação rápida permite o acesso rápido às informações, aspecto importante para a fluência de leitura, por meio do envolvimento de diversos recursos cognitivos^(10)^. Quando as informações são acessadas de forma rápida e precisa no léxico mental, mais recursos cognitivos e atencionais estarão disponíveis para a leitura^(11)^.

Os estudos que investigam a interação das habilidades linguísticas e cognitivas com o processamento fonológico mostram a relação de reciprocidade^(2,12)^. Assim a apropriação e o aumento na proficiência da leitura e da escrita também impulsionam e podem explicar o desenvolvimento do processamento fonológico para níveis mais elevados^(2,12)^. No entanto, ainda são escassos os estudos que investigam o desempenho de adolescentes no processamento fonológico como variável resposta e explicado pelas habilidades linguístico-cognitivas.

Assim, este estudo teve por objetivo caracterizar o processamento fonológico em adolescentes e identificar habilidades linguísticas e funções cognitivas que o influenciam nessa faixa etária.

## MÉTODO

Este estudo, com delineamento transversal observacional analítico, foi aprovado pelo Comitê de Ética em Pesquisa da Universidade Federal de Minas Gerais sob o Parecer nº 1.722.230. Os adolescentes e seus responsáveis assinaram os termos de autorização para participar do estudo.

### Participantes

A amostra foi não probabilística, de conveniência. Os adolescentes do Ensino Fundamental II de duas escolas públicas de Belo Horizonte foram convidados para participar do estudo, sendo que 110 adolescentes apresentaram interesse em compor a amostra do estudo. Foram selecionados aqueles cujos pais relataram, no questionário de anamnese, desenvolvimento típico. Como critério de exclusão adotou-se a presença de alterações neurológicas, psiquiátricas, cognitivas e de aprendizagem evidentes, relatadas durante a aplicação do questionário de anamnese, alterações auditivas e visuais não corrigidas e a não conclusão da testagem. Após a análise dos questionários de anamnese, foram excluídos quatro adolescentes por apresentarem diagnósticos dos transtornos do desenvolvimento referidos pelos pais. Assim, a amostra inicial foi composta por 106 adolescentes com desenvolvimento típico. Ao longo da pesquisa, 23 adolescentes abandonaram o estudo e não concluíram as avaliações. Portanto, a amostra final foi composta por 83 adolescentes de ambos os sexos, regularmente matriculados do sexto ao nono ano. Como foram selecionados os adolescentes regulares nos anos escolares do Ensino Fundamental II, a faixa etária da amostra se situou de 11 a 16 anos.

### Instrumentos

- Questionário de anamnese - elaborado pelas pesquisadoras com questões referentes ao histórico de saúde e do desenvolvimento do adolescente.
- Teste de consciência fonológica da Bateria de Avaliação da Linguagem Escrita e seus Distúrbios – BALESC^(13,14)^: o teste é composto por tarefas de manipulação silábica e fonêmica. A literatura evidencia que as experiências linguísticas e acadêmicas presentes ao ingressar na escola favorecem as aquisições no nível silábico da consciência fonológica, possibilitando, nos anos escolares iniciais, bom desempenho em tal nível, tornando-o internalizado no ensino fundamental. Além disso, como a consciência fonêmica apresenta maior relação com a leitura^(5)^, optou-se por utilizar somente as tarefas de manipulação fonêmica, sendo elas: segmentação, subtração e inversão. Os estímulos do teste foram gravados para evitar interferências.
- Prova de velocidade de nomeação automática RAN – *rapid automatized naming* ^(15)^: foram utilizadas as pranchas com os estímulos visuais letras e objetos dispostos de forma aleatória em dez colunas e cinco linhas.
- Instrumento de Avaliação Neuropsicológica Breve – NEUPSILIN^(16)^: para avaliar atenção foram aplicadas as provas de contagem inversa e de repetição de dígitos. A memória foi avaliada por meio de provas de span de dígitos e de palavras, reconhecimento, evocação imediata e tardia, memória semântica de longo prazo, memória visual de curto prazo e memória prospectiva. Para avaliar a linguagem oral, foram realizadas as tarefas de nomeação, repetição, compreensão, processamento de inferências e de linguagem automática. As funções executivas foram avaliadas por meio da resolução de problemas orais e de fluência verbal. Conforme a correção proposta pelos autores do instrumento, o resultado da memória operacional, da memória verbal episódica-semântica, da atenção e da linguagem oral foram apresentados também de forma qualitativa, classificados como adequado ou inadequado segundo os padrões referidos pelo teste.
- Teste de fluência e compreensão de leitura textual: Foi utilizado o instrumento proposto por Gentilini et al.^(17)^, composto por um texto e dez questões literais e inferenciais. O instrumento foi elaborado para avaliar adolescentes do ensino fundamental II. A construção do instrumento envolveu diversas etapas, dentre elas, a seleção e análise por juízes especialistas e análise computacional, de forma que cumprisse os critérios para a avaliação linguística de adolescentes.

### Procedimentos

Os participantes foram avaliados em uma sala silenciosa, na própria escola, em sessões de 30 minutos por dia, com tempo médio total de duas horas. No primeiro momento foi enviado, aos pais, juntamente com os termos de autorização para participar do estudo, o questionário de anamnese e recolhido posteriormente, para auxiliar na seleção dos adolescentes de acordo com critérios de inclusão e exclusão.

Posteriormente, os adolescentes selecionados foram submetidos aos testes de descritos anteriormente, seguindo as normas de aplicações orientadas por seus autores nas pesquisas e manuais publicados. Somente a avaliação de leitura foi aplicada em coletivo.

### Análise dos dados

Os dados foram inseridos em planilha no Excel®. Na análise descritiva das variáveis qualitativas, foram utilizadas as frequências absoluta e relativa, enquanto na descrição das variáveis quantitativas foram utilizadas medidas de posição, tendência central e dispersão.

Para verificar o efeito das variáveis cognitivas e linguísticas sobre o processamento fonológico, utilizou-se uma regressão linear com erros padrões robustos para a matriz de covariância dos coeficientes estimados e o estimador robusto HC (*heteroskedasticity and autocorrelacion consistent*) na estimativa da matriz de covariância.

Para a seleção de variáveis, foi utilizado o método *Stepwise* (*Forward* e *Backward*). Dessa forma, usando o método *Forward*, foi feita uma análise univariada, que consistiu no ajuste de uma regressão linear com erros padrões robustos, para cada uma das variáveis separadamente. As variáveis com valor-p inferior a 0,25 foram selecionadas para a análise multivariada. Na análise multivariada, foi ajustado um modelo com todas as variáveis selecionadas. Em seguida, verificou-se a presença de multicolinearidade entre elas. Para tanto, foi utilizada a estatística VIF (V*ariance Inflation Factor*), sendo que as variáveis com VIF superior a cinco foram retiradas do modelo. Em seguida, foi aplicado o método *Backward*, procedimento usado de retirar, por vez, a variável de maior valor-p, o qual foi repetido até que restassem no modelo somente as variáveis significativas. Para o método *Backward*, foi adotado o nível de significância de 5%. Foi utilizado o software R (versão 3.4.3) nas análises.

As variáveis fluência leitora, compreensão textual, atenção, memória verbal episódico-semântica, funções executivas e tarefas de linguagem oral foram consideradas explicativas para as variáveis respostas: tempo de nomeação em objetos e letras, memória operacional e consciência fonológica, que também foram explicativas umas das outras nos modelos da análise de regressão.

## RESULTADOS

As medidas de tendência central e de dispersão das variáveis clínicas foram apresentadas nas Figuras 1 e 2.

**Figura 1:** Medidas de tendência central e dispersão das tarefas do Processamento Fonológico

**Legenda**: cvc = consoante-vogal-consoante; ccv = consoante-consoante-vogal; seg = segundos

**Figura 2:** Medidas de tendência central e dispersão das variáveis cognitivas e linguísticas

**Legenda**: min = minute; M. = memória

No que se refere à classificação do desempenho dos participantes nas tarefas da avaliação neuropsicológica, verificou-se que 82,7% (*n* = 62) apresentaram desempenho adequado em memória operacional, 87,8% (*n* = 65) em atenção, 65,3% (*n* = 49) em memória verbal episódico-semântica e 85,3% (*n* = 64) nas tarefas de linguagem oral. A linguagem oral foi avaliada por meio das tarefas de nomeação, repetição, compreensão e processamento de inferências e de linguagem automática. Assim, o termo “linguagem oral adequada” foi utilizado quando o escore geral estava dentro da faixa de normalidade determinada pelo teste.

### Fatores associados ao tempo de nomeação de objetos e letras

A Tabela 1 apresenta os resultados da análise univariada entre os fatores cognitivos e linguísticos estudados e a nomeação rápida de objetos e letras. De maneira geral a nomeação rápida apresentou associação estatisticamente significante com quase todas as variáveis analisadas.

**Tabela 1:** Fatores associados à nomeação rápida de objetos e letras - análise univariada

**Variável**	**Nomeação de objetos**				**Nomeação de letras**			
	***β***	**E.P.** 1	**I.C. - 95%^1^**	**Valor- *p* ^1^**	***β***	**E.P.^1^**	**I.C. - 95%^1^**	**Valor- *p* ^1^**
Memória operacional	-0,97	0,34	[-1,64; -0,30]	**0,005**	-0,11	0,10	[-0,30; 0,09]	0,275
Memória operacional (adeq.)	-	-	-	-	-	-	-	-
Memória operacional (inadeq.)	12,37	4,93	[2,71; 22,04]	**0,012**	2,41	1,22	[0,02; 4,80]	**0,048**
Consciência fonológica	-0,62	0,16	[-0,93; -0,31]	**< 0,001**	-0,28	0,06	[-0,39; -0,16]	**< 0,001**
Nomeação de letras	1,45	0,25	[0,97; 1,93]	**< 0,001**	**-**	**-**	**-**	**-**
Nomeação de objetos	-	-	-	**-**	0,24	0,04	[0,16; 0,32]	**< 0,001**
Fluência de leitura	-0,12	0,03	[-0,18; -0,06]	**< 0,001**	-0,05	0,01	[-0,07; -0,03]	**< 0,001**
Compreensão textual	-2,01	0,52	[-3,03; -0,98]	**< 0,001**	-0,73	0,18	[-1,09; -0,37]	**< 0,001**
Memória verbal episódico-semântica	-0,33	0,42	[-1,14; 0,49]	0,430	-0,24	0,15	[-0,54; 0,06]	0,124
M.verbal episódico-semântica (adeq.)	-	-	-	-	-	-	-	-
M. verbal episódico-semântica (inadeq.)	2,80	2,96	[-3,00; 8,61]	0,343	2,55	1,25	[0,10; 5,00]	**0,042**
Tarefas de linguagem oral	-4,49	1,73	[-7,89; -1,09]	**0,010**	-1,27	0,57	[-2,38; -0,16]	**0,025**
Tarefas de linguagem oral (adeq.)	-	-	-	-	-	-	-	-
Tarefas de linguagem oral (inadeq.)	8,59	5,38	[-1,96; 19,13]	0,111	2,43	1,48	[-0,48; 5,34]	0,101
Tarefas de funções executivas	-2,44	0,61	[-3,64; -1,24]	**< 0,001**	-0,72	0,26	[-1,23; -0,21]	**0,006**
Atenção	-0,62	0,33	[-1,28; 0,03]	**0,063**	-0,16	0,08	[-0,31; -0,01]	**0,037**
Atenção (adeq.)	-	-	-	-	-	-	-	-
Atenção (inadeq.)	9,62	5,77	[-1,68; 20,92]	0,095	1,82	1,22	[-0,58; 4,21]	0,137

1 Calculado utilizando o estimador HC;

**Legenda**: *β* = coeficiente da regressão; E.P. = erro padrão; I.C = intervalo de confiança; adeq. = adequada; inadeq. = inadequada; M. = memória; *p*<0,05

O modelo final da regressão multivariada (Tabela 2), apontou que cada um ponto a mais no escore da memória operacional leva a uma redução média de -0,72 segundos do tempo de nomeação de objetos e que o aumento de 1 segundo na nomeação das letras leva a um aumento médio de 1,05 segundos no tempo de nomeação de objetos. Em relação ao modelo final da análise multivariada para a nomeação de letras (Tabela 2), observa-se que cada um ponto que aumenta o desempenho em consciência fonológica e cada aumento de um palavra no total de palavras lidas por minuto leva à redução de -0,11 e 0,02 segundos, respectivamente, no tempo de nomeação de letras. Além disso, a cada aumento de 1s no tempo de nomeação dos objetos ocorre em média um aumento de 0,18s no tempo de nomeação de letras.

**Tabela 2:** Fatores associados à nomeação rápida de objetos e letras - análise multivariada

**Variável**	**Nomeação de objetos**								**Nomeação de letras**							
	**Modelo inicial**				**Modelo final**				**Modelo inicial**				**Modelo final**			
	***β***	**E.P.** 1	**I.C. - 95%^1^**	**Valor- *p* ^1^**	***β***	**E.P.^1^**	**I.C. - 95%^1^**	**Valor- *p* ^1^**	***β***	**E.P.^1^**	**I.C. - 95%^1^**	**Valor- *p* ^1^**	***β***	**E.P.^1^**	**I.C. - 95%^1^**	**Valor- *p* ^1^**
Memória operacional	-0,80	0,33	[-1,44; -0,15]	**0,015**	-0,72	0,26	[-1,24; -0,21]	**0,006**								
Consciência fonológica	0,12	0,17	[-0,21; 0,44]	0,489	-	-	-	-	-0,12	0,08	[-0,27; 0,03]	0,105	-0,11	0,05	[-0,22; -0,01]	**0,027**
Memória operacional (adeq.)									-	-	-	-	-	-	-	-
Memória operacional (inadeq.)									-0,47	1,21	[-2,85; 1,91]	0,698	-	-	-	-
Memória verbal episódica-semântica									-0,19	0,15	[-0,48; 0,09]	0,182	-	-	-	-
Memória verbal episódico-semântica (adeq.)	-	-	-	-	-	-	-	-								
Memória verbal episódico-semântica (inadeq.)	-4,30	2,53	[-9,25; 0,66]	0,089	-	-	-	-								
Nomeação de letras	1,11	0,23	[0,65; 1,56]	**<0,001**	1,05	0,20	[0,65; 1,44]	**<0,001**								
Nomeação de objetos									0,18	0,05	[0,08; 0,28]	**<0,001**	0,18	0,04	[0,10; 0,27]	**<0,001**
Fluência de leitura	-0,04	0,02	[-0,08; 0,00]	**0,032**	-0,04	0,02	[-0,07; 0,00]	**0,051**	-0,02	0,01	[-0,04; 0,00]	**0,016**	-0,02	0,01	[-0,04; 0,00]	**0,020**
Compreensão textual	-0,77	0,44	[-1,62; 0,09]	0,078	-0,73	0,38	[-1,48; 0,03]	**0,058**	-0,10	0,22	[-0,53; 0,34]	0,667	-	-	-	-
Tarefas de linguagem oral	-1,51	0,99	[-3,46; 0,43]	0,127	-	-	-	-	-0,06	0,46	[-0,96; 0,84]	0,897	-	-	-	-
Tarefas de funções executivas	0,04	0,62	[-1,18; 1,25]	0,951	-	-	-	-	-0,01	0,26	[-0,53; 0,50]	0,958	-	-	-	-
Atenção	0,10	0,22	[-0,34; 0,53]	0,668	-	-	-	-	0,11	0,10	[-0,08; 0,31]	0,244	-	-	-	-
R^2^	46,21%				48,16%				37,21%				38,60%			
VIF Máximo	1,79				1,36				1,67			

1 Estimador HC;

**Legenda**: *β* = coeficiente da regressão; E.P = erro padrão; I.C = intervalo de confiança; adeq. = adequada; inadeq. = inadequada, *p*<0,05

### Fatores associados à memória operacional

A Tabela 3 apresenta os resultados da análise univariada dos fatores cognitivos e linguísticos estudados e a memória operacional (contínua e categórica). Verificou-se associação estatisticamente significante entre a memória operacional e as seguintes variáveis: consciência fonológica (contínua), nomeação de objetos (contínua e categórica), compreensão textual (categórica), memória verbal episódico-semântica (contínua), linguagem oral (contínua), funções executivas (contínua e categórica) e atenção (contínua e categórica).

**Tabela 3:** Fatores associados à pontuação na memória operacional e à classificação do desempenho - análise univariada

**Variável**	**Pontuação**				**Classificação do desempenho**				
	***β***	**E.P.** 1	**I.C. - 95%^1^**	**Valor- *p* ^1^**	***β***	**E.P.^1^**	**I.C. - 95%^1^**	**Valor- *p* ^1^**	**O.R.**
Nomeação de letras	-0,12	0,11	[-0,34; 0,1]	0,297	0,10	0,06	[-0,01; 0,21]	0,074	1,11
Consciência fonológica	0,17	0,08	[0,01; 0,33]	**0,041**	-0,06	0,04	[-0,14; 0,01]	0,113	0,94
Nomeação de objetos	-0,17	0,05	[-0,27; -0,07]	**0,001**	0,08	0,02	[0,03; 0,12]	**0,001**	1,08
Fluência de leitura	0,02	0,01	[-0,01; 0,04]	0,190	-0,01	0,01	[-0,03; 0,01]	0,274	0,99
Compreensão textual	0,41	0,22	[-0,02; 0,83]	0,060	-0,26	0,13	[-0,51; -0,01]	**0,043**	0,77
Memória verbal episódico-semântica	0,50	0,13	[0,25; 0,74]	**<0,001**	-0,17	0,10	[-0,37; 0,04]	0,107	0,85
Memória verbal episódico-semântica (adeq.)	-	-	-	-	-	-	-	-	-
Memória verbal episódico-semântica (inadeq.)	-3,28	1,15	[-5,54; -1,02]	**0,004**	0,97	0,62	[-0,25; 2,19]	0,118	2,64
Tarefas de linguagem oral	1,70	0,82	[0,09; 3,3]	**0,039**	-0,69	0,38	[-1,44; 0,06]	0,072	0,50
Tarefas de linguagem oral (adeq.)	-	-	-	-	-	-	-	-	-
Tarefas de linguagem oral (inadeq.)	-3,14	2,36	[-7,76; 1,48]	0,183	2,28	0,73	[0,86; 3,7]	**0,002**	9,77
Tarefas de funções executivas	1,35	0,25	[0,87; 1,83]	**<0,001**	-0,49	0,18	[-0,84; -0,14]	**0,006**	0,61
Atenção	0,52	0,10	[0,33; 0,72]	**<0,001**	-0,19	0,05	[-0,28; -0,1]	**<0,001**	0,83
Atenção (adeq.)	-	-	-	-	-	-	-	-	-
Atenção (inadeq.)	-6,43	1,98	[-10,31; -2,55]	**0,001**	1,60	0,76	[0,11; 3,1]	**0,035**	4,98

1 Calculado utilizando o estimador HC;

**Legenda**: *β* = coeficiente da regressão; E.P. = erro padrão; I.C = intervalo de confiança; O.R. = *oddsratio*; adeq. = adequado; inadeq. = inadequado; *p*<0,05

O modelo final da regressão multivariada (Tabela 4) apontou que a cada aumento de 1 segundo no tempo de nomeação dos objetos ocorre em média uma redução de 0,09 no escore de memória operacional. O acréscimo de uma unidade no escore de memória verbal episódico-semântica, funções executivas e atenção leva ao aumento respectivo de 0,33, 0,83 e 0,34 pontos no escore da memória operacional. Apesar de no modelo final o VIF ter sido baixo, a variável compreensão textual estava correlacionada com quase todas as variáveis explicativas do modelo final, como atenção, funções executivas, memória verbal episódico-semântico e tempo de nomeação de objetos. Um modelo com variáveis explicativas correlacionadas leva à ocorrência de multicolinearidade o que acarreta a inversão do sinal do coeficiente estimado (*β*). Dessa forma, foi necessário retirar a variável compreensão textual do modelo final.

**Tabela 4:** Fatores associados à pontuação na memória operacional e à classificação do desempenho - análise multivariada

**Variável**	**Pontuação**								**Classificação do desempenho**									
	**Modelo inicial**				**Modelo final**				**Modelo inicial**				**Modelo final**					
	***β***	**E.P.** 1	**I.C. - 95%^1^**	**Valor- *p* ^1^**	***β***	**E.P.^1^**	**I.C. - 95%^1^**	**Valor- *p* ^1^**	***β***	**E.P.^1^**	**I.C. - 95%^1^**	**Valor- *p* ^1^**	***β***	**E.P.^1^**	**I.C. - 95%^1^**	**Valor- *p* ^1^**	**O.R^1^**	**I.C. - 95%^1^(O.R)**
Consciência fonológica	-0,04	0,07	[-0,18; 0,09]	0,397	-	-	-	-	0,05	0,05	[-0,05; 0,15]	0,294	-	-	-	-	-	-
Nomeação de letras									-0,06	0,11	[-0,28; 0,15]	0,567	-	-	-	-	-	-
Nomeação de objetos	-0,12	0,04	[-0,19; -0,05]	**0,001**	-0,09	0,04	[-0,17; -0,01]	**0,034**	0,08	0,04	[0,00; 0,16]	**0,062**	0,06	0,03	[0,01; 0,12]	**0,028**	1,07	[1,01; 1,13]
M. verbal episódico-semântica	0,32	0,09	[0,14; 0,51]	**<0,001**	0,33	0,09	[0,14; 0,51]	**<0,001**										
Fluência de leitura	-0,01	0,01	[-0,03; 0,01]	0,369	-	-	-	-	0,00	0,01	[-0,03; 0,02]	0,803	-	-	-	-	-	-
Compreensão textual	-0,31	0,17	[-0,64; 0,03]	**0,060**	-	-	-	-	0,03	0,19	[-0,34; 0,41]	0,861	-	-	-	-	-	-
Tarefas de linguagem oral	0,50	0,54	[-0,57; 1,56]	0,344	-	-	-	-	-0,39	0,48	[-1,34; 0,56]	0,418	-	-	-	-	-	-
Tarefas de funções executivas	1,00	0,26	[0,48; 1,51]	**<0,001**	0,83	0,25	[0,34; 1,32]	**0,001**	-0,53	0,35	[-1,21; 0,15]	0,126	-0,43	0,26	[-0,95; 0,09]	0,104	0,65	[0,39; 1,09]
Atenção	0,37	0,08	[0,21; 0,54]	**<0,001**	0,34	0,08	[0,2; 0,49]	**<0,001**	-0,17	0,06	[-0,30; -0,05]	**0,005**	-0,16	0,05	[-0,25; -0,07]	**0,001**	0,85	[0,78; 0,93]
R^2^	45,39%				43,47%				42,32%				39,64%					
VIF Máximo	1,59				1,17				2,06				1,00					

1 Calculado utilizando o estimador HC;

**Legenda**: *β* = coeficiente da regressão; E.P. = erro padrão; I.C = intervalo de confiança; O.R. = *oddsratio*; M. verbal episódico-semântica = memória verbal episódico-semântica; *p*<0,05

Em relação ao modelo final de regressão multivariada para a classificação do desempenho na memória operacional (Tabela 4), observa-se que o aumento em um segundo no tempo de nomeação de objetos aumenta em 1,07 vezes as chances de a memória operacional ser classificada como inadequada. Em contrapartida, a cada um ponto que aumenta no escore da atenção diminui em 15% chance de a memória operacional ser classificada como inadequada.

### Fatores associados à consciência fonológica

Os resultados da análise de regressão uni e multivariada para consciência fonológica são apresentados na Tabela 5. Observa-se associação estatisticamente significante entre a consciência fonológica e o tempo de nomeação de letras e objetos, memória operacional, linguagem oral, fluência de leitura, compreensão de leitura, funções executivas e atenção.

**Tabela 5:** Fatores associados à consciência fonológica - análise uni e multivariada

**Variável**	**Análise univariada**				**Análise multivariada**								
	***β***	**E.P.** 1	**I.C. - 95%^1^**	**Valor- *p* ^1^**	**Modelo inicial**				**Modelo final**				
					***β***	**E.P.^1^**	**I.C. - 95%^1^**	**Valor- *p* ^1^**	***β***	**E.P.^1^**	**I.C. - 95%^1^**	**Valor- *p* ^1^**	
Nomeação de letras	-0,50	0,12	[-0,74; -0,27]	**< 0,001**	-0,21	0,13	[-0,47; 0,06]	0,125	-0,37	0,10	[-0,57; -0,17]	**< 0,001**	
Memória operacional (adeq.)	-	-	-	-									
Memória operacional (inadeq.)	-2,82	1,65	[-6,06; 0,42]	0,088									
Memória operacional	0,28	0,11	[0,07; 0,50]	**0,010**	-0,12	0,16	[-0,44; 0,19]	0,444	-	-	-	-	
Nomeação de objetos	-0,18	0,05	[-0,28; -0,09]	**< 0,001**	0,00	0,06	[-0,11; 0,12]	0,947	-	-	-	-	
Fluência de leitura	0,05	0,01	[0,03; 0,07]	**< 0,001**	0,02	0,01	[-0,01; 0,04]	0,198	-	-	-	-	
Compreensão textual	1,06	0,23	[0,62; 1,51]	**< 0,001**	0,43	0,24	[-0,05; 0,91]	0,077	-	-	-	-	
Memória verbal episódico-semântica	0,09	0,17	[-0,25; 0,43]	0,592									
Memória verbal episódico-semântica (adeq.)	-	-	-	-									
Memória verbal episódico-semântica (inadeq.)	-0,03	1,52	[-3,02; 2,95]	0,983									
Tarefas de linguagem oral	1,89	0,64	[0,64; 3,15]	**0,003**	0,65	0,64	[-0,60; 1,90]	0,310	-	-	-	-	
Tarefas de linguagem oral (adeq.)	-	-	-	-									
Tarefas de linguagem oral (inadeq.)	-4,28	2,40	[-8,97; 0,42]	0,074									
Tarefas de funções executivas	1,40	0,40	[0,62; 2,19]	**< 0,001**	0,71	0,43	[-0,13; 1,55]	0,097	0,87	0,42	[0,04; 1,70]	**0,039**	
Atenção	0,50	0,09	[0,32; 0,69]	**< 0,001**	0,34	0,12	[0,11; 0,57]	**0,004**	0,37	0,10	[0,17; 0,57]		**< 0,001**
Atenção (adeq.)	-	-	-	-									
Atenção (inadeq.)	-6,04	1,85	[-9,67; -2,42]	**0,001**									
R^2^					27,38%				26,36%				
VIF Máximo					2,09				1,11				

1 Estimador HC; **Legenda**: *β* = coeficiente da regressão; E.P = erro padrão; I.C = intervalo de confiança; adeq. = adequada; inadeq. = inadequada; *p*<0,05

O modelo final da regressão multivariada apontou que o aumento de um segundo no tempo na nomeação de letras reduz -0,37 pontos no escore de consciência fonológica. No entanto, a cada uma unidade que aumenta no escore de funções executivas e atenção ocorre em média um acréscimo de 0,87 e 0,37, respectivamente, no desempenho em consciência fonológica.

## DISCUSSÃO

A literatura^(3)^ apresenta a importância do processamento fonológico no aprendizado inicial da leitura e da escrita em crianças. Porém, ainda há lacunas no conhecimento sobre o desempenho de indivíduos mais velhos (que já alcançaram certo nível de desenvolvimento cognitivo e de aprendizado) no processamento fonológico e os fatores que interferem em tal desempenho. Assim, buscou-se neste estudo caracterizar as habilidades do processamento fonológico em adolescentes e apontar quais fatores cognitivos e linguísticos influenciam o desenvolvimento dessas habilidades.

Em relação ao desempenho cognitivo, os adolescentes selecionados não apresentavam queixas de alterações de linguagem ou cognitiva, portanto, eram considerados adolescentes com desenvolvimento típico. Assim, esperava-se que o desempenho fosse adequado, como foi observado na maioria da amostra. Neste estudo, os adolescentes apresentaram desempenho semelhante ao dos adultos jovens com maior grau de escolaridade de pesquisa anterior^(18)^. No entanto, não foi observado efeito de teto, como ocorreu no estudo em questão, o que sugere que o desempenho cognitivo dos adolescentes está a caminho do ápice do desenvolvimento.

Os dados descritivos da nomeação rápida mostraram que o menor tempo de nomeação foi em letras e o maior tempo de nomeação em objetos e corroborou os dados descritos em estudos anteriores^(12)^. Segundo a literatura, o conhecimento de letras e dígitos é automatizado rapidamente, o que confere agilidade na nomeação e na redução do tempo gasto^(6)^. Quanto à nomeação de cores e objetos, os estudos mostram que os conceitos e as informações semânticas são acessados anteriormente à nomeação, além dos estímulos possuírem maior extensão e complexidade articulatória, o que eleva o tempo gasto para concluir a prova^(12)^.

Em relação ao desempenho em memória operacional, a maioria dos participantes, por revelarem desenvolvimento típico e estarem na faixa etária em que se observa a ampliação da memória operacional, apresentaram desempenho adequado, de acordo com os escores propostos no teste NEUPSILIN^(16)^. Sabe-se que o aumento da idade e do ano escolar amplia as capacidades da memória operacional possibilitando a manipulação de maior quantidade de informações simultaneamente^(8,9)^.

O desempenho dos adolescentes em consciência fonológica foi melhor na tarefa de subtração consoante-vogal-consoante (CVC), seguindo-se as tarefas de inversão e subtração consoante-consoante-vogal (CCV). Na tarefa de segmentação fonêmica, eles apresentaram a menor média de acertos, demonstrando ser esta a de maior grau de dificuldade para a amostra avaliada. Em uma pesquisa, ao avaliar alunos do ensino fundamental, do 1º ao 5º ano com tarefas de segmentação e subtração fonêmica, constatou melhor desempenho nas provas de subtração CVC e CCV^(19)^. Na prova de segmentação fonêmica, encontrou-se menor média de acertos. No estudo, não foi realizada a tarefa de inversão fonêmica.

Em outro estudo^(20)^, em que foram analisadas três pesquisas com adolescentes e adultos, foi verificado número baixo de acertos na tarefa de segmentação, mesmo para indivíduos proficientes na leitura. De acordo com o estudo em questão, após o desenvolvimento da leitura pode haver menor necessidade de analisar a palavra a partir da sua estrutura fonológica, assim ocorreria o declínio em tal habilidade por desuso. A segunda hipótese apresentada sugere que ao adquirir a proficiência na leitura, o indivíduo adquire outras estratégias, como a análise da palavra no nível ortográfico. Por fim, o estudo indica que é necessário o treinamento explícito para o desenvolvimento da consciência fonêmica. Naturalmente a atenção do indivíduo é direcionada para estruturas sonoras maiores, como a rima e a aliteração, a percepção de fonemas é desenvolvida com treinamento. Portanto, indivíduos pouco estimulados apresentariam menor nível de consciência fonêmica e essa não seria uma condição que impediria o alcance de níveis elevados de leitura.

É importante destacar, também, que os fonemas são unidades abstratas destituídas de significado. Durante a segmentação fonêmica, o adolescente manipula os fonemas sem o apoio da informação acústica da palavra e direciona a atenção para a sua forma estrutural, e não para o significado. Isso eleva o grau de dificuldade da tarefa, justificando o baixo desempenho dos adolescentes^(19)^. Não se observou efeito de teto entre os adolescentes, o que sugere que esse público está em fase de aprimoramento da consciência fonêmica e que a dificuldade na manipulação de fonemas isolados persistia na amostra estudada.

Os resultados da análise de regressão linear apontaram que o processamento fonológico foi influenciado por habilidades linguísticas e funções cognitivas, além de haver interferência mútua entre as habilidades do processamento fonológico. Constatou-se que a memória operacional, o tempo de nomeação de letras e objetos, a consciência fonológica e as funções executivas foram mais significativas para explicar o desempenho dos adolescentes no processamento fonológico.

Assim, a nomeação rápida de objetos foi mais influenciada pela memória operacional e a nomeação de letras. Já o tempo de nomeação de letras foi mais influenciado pelo desempenho em consciência fonológica e tempo de nomeação de objetos. O desempenho dos adolescentes na memória operacional e na consciência fonológica foi mais bem explicado pelas funções executivas.

A nomeação rápida de estímulos visuais demanda o envolvimento de diversos processos atencionais, de memória, perceptuais, conceituais, lexicais e articulatórios^(21)^. Todos esses processos funcionam de forma harmônica para conferir agilidade e precisão em todas as tarefas de nomeação, o que sugere a influência do desempenho de uma tarefa de nomeação sobre a outra. Observou-se, na amostra a associação entre os tempos de nomeação de objetos e letras. Assim, indivíduos que demandam maior tempo na nomeação de estímulos não alfanuméricos tendem a apresentar maior tempo na nomeação de estímulos alfanuméricos, sendo que a relação inversa também está presente.

O tempo de nomeação de objetos também foi influenciado pela memória operacional. Então, à medida que aumenta o escore da memória operacional, o tempo de nomeação de objetos reduz. Devido às características dos estímulos presentes no teste RAN, apurou-se que a nomeação de objetos apresenta uma base semântica e maior extensão e complexidade fonológica do que a nomeação de letras, que apresenta base fonológica e menor extensão e complexidade articulatória^(22)^. Além desses fatos, apesar de ambas as atividades serem automáticas, soma-se o de a característica da nomeação de letras ser um processo automatizado primeiro em relação à nomeação de objetos^(22)^. Assim, a nomeação de objetos exigiu mais dos recursos da memória operacional para que os estímulos fossem resgatados e mantidos de forma acessível na memória enquanto eram nomeados, o que não se observou de forma significativa na nomeação de letras, que demandou menos esforços e atuação do processo mnemônico^(6,23)^.

De forma inversa, à medida que aumenta o tempo de realização da tarefa de nomeação, tende-se a reduzir o escore de memória operacional, aumentando as chances de o desempenho ser classificado como inadequado para alguns adolescentes. Como a nomeação de objetos necessita de recursos da memória operacional^(21)^, o aumento no tempo de execução da prova de nomeação pode ser reflexo de prejuízos nos recursos mnemônicos. Assim, verificou-se influência mútua entre nomeação rápida de objetos e memória operacional.

Outro processo relevante para a nomeação rápida foi a consciência fonológica. Observou-se nos adolescentes avaliados que, o melhor desempenho dos adolescentes em consciência fonológica, contribuiu para a agilidade na nomeação de letras. Em tal tarefa a manipulação fonológica é exigida durante toda a prova, por isso o manejo das representações fonológicas com facilidade mostrou-se importante para conferir maior velocidade na nomeação^(24)^.

De acordo com os resultados do presente estudo, os adolescentes que executaram as tarefas de nomeação em menor tempo apresentaram melhor desempenho em outras tarefas. Assim, ressalta-se que a agilidade na execução de tarefas é um fator relevante para o desempenho cognitivo e linguístico do indivíduo.

As funções executivas, responsáveis por regular o processo de informação e comportamento^(25)^, também foram significativas para explicar o desempenho dos adolescentes na consciência fonológica e na memória operacional, contribuindo para elevar os escores de tais habilidades.

Devido ao fato de controlarem as ações^(25)^, as funções executivas mostram-se relevantes durante as tarefas de manipulação fonêmica. A grande importância desta habilidade fica ainda mais evidente em estudos com indivíduos com TDAH. Segundo os pesquisadores, tais indivíduos apresentam alterações em linguagem exatamente em decorrência do déficit nas funções executivas, levando ao prejuízo no processamento fonológico^(6)^. Os autores observaram que alunos com TDAH apresentam desempenho inferior em consciência fonológica e memória operacional quando comparados com alunos típicos. Portanto, os dados do presente estudo corroboram a literatura ao apontar que as funções executivas são relevantes para a execução da tarefa de consciência fonológica.

Quanto à associação das funções executivas com a memória operacional, no presente estudo foram utilizadas as tarefas de fluência verbal e de resolução de problemas para avaliar a função executiva. Essas tarefas demandam a evocação e manipulação da informação para serem concluídas e ao relacionar palavras com agilidade ou resolver problemas com facilidade há menor sobrecarga no sistema mnemônico^(25)^. Por isso, o bom desempenho das funções executivas se correlaciona positivamente com a memória operacional. Os resultados mostraram que dentre os adolescentes com bom desempenho em função executiva existe a tendência de reduzir as chances de serem classificados com desempenho inadequado na memória operacional.

Outra função cognitiva que influenciou o desempenho do processamento fonológico foi a atenção, que se faz necessária para focar no estímulo relevante e inibir os distratores. Assim, a capacidade de armazenamento depende da manutenção do foco no estímulo necessário, o que sugere uma relação entre a atenção e a memória^(9)^.

Em concordância com essa hipótese, em outro estudo os pesquisadores verificaram que na presença de alteração na atenção a memória também se apresenta comprometida^(6)^. Entre os adolescentes avaliados no presente estudo, a atenção revelou-se como um dos fatores determinantes para o desempenho da memória operacional

Vários estudos investigam o processamento fonológico em crianças. No entanto, há a necessidade de expandir as pesquisas com adolescentes, a fim de aprofundar os conhecimentos sobre essas habilidades nesse público-alvo e as variáreis que as influenciam, de forma a colaborar para a prática clínica e a seleção e elaboração de instrumentos de avaliação adequados para adolescentes.

A forma de seleção da amostra desta pesquisa constituiu uma limitação para a generalização dos dados. No entanto, apesar da amostra ser pequena pode ocorrer dos dados desse estudo se reproduzirem em uma amostra maior, devido aos baixos valores de *β* e de erro-padrão obtidos nas análises. Sugere-se a continuação de estudos com adolescentes a fim de aprofundar as investigações realizadas.

Este estudo mostrou que o clínico deve estar atento às habilidades linguísticas e às funções cognitivas que influenciam o processamento fonológico, visto que podem impactar no processo terapêutico.

## CONCLUSÃO

Pode-se concluir que, para os adolescentes avaliados, a nomeação automática de letras foi realizada em menor tempo em relação à nomeação automática de objetos e houve menor média de acertos na tarefa de segmentação fonêmica. As habilidades do processamento fonológico se relacionaram entre si. As funções executivas e a atenção foram mais significativas para explicar as habilidades do processamento sonoro da informação, em comparação à leitura e à memória verbal episódico-semântica que se mostraram menos relevantes.
